# The Effect of Iodine Status on the Risk of Thyroid Nodules: A Cross-Sectional Study in Zhejiang, China

**DOI:** 10.1155/2020/3760375

**Published:** 2020-08-18

**Authors:** Xiaoming Lou, Xiaofeng Wang, Zhifang Wang, Guangming Mao, Wenming Zhu, Yuanyang Wang, Xuejiao Pan, Zhijian Chen, Zhe Mo

**Affiliations:** Department of Environmental Health, Zhejiang Provincial Center for Disease Prevention and Control, 3399 Binsheng Road, Hangzhou 310051, Zhejiang, China

## Abstract

**Objective:**

The aim of this study was to explore whether iodine nutrition is associated with the risk of thyroid nodules among adult population in Zhejiang Province, China.

**Methods:**

A cross-sectional study was conducted in the general population aged 18 years or older. A total of 2,710 subjects received physical examination, questionnaires, and thyroid ultrasonography. Urinary iodine concentration (UIC) and thyroid hormone levels were measured and documented for each subject. 4 multiple logistic regression models adjusted for other risk factors were applied to analyze the association between iodine nutrition and thyroid nodules.

**Results:**

The prevalence of thyroid nodules was 15.5% among all adults. As indicated by all 4 models, subjects with UIC varying from 200 *μ*g l^−1^ to 399 *μ*g l^−1^ had lower risk of thyroid nodules compared with those with relatively low UIC (<100 *μ*g l^−1^), with approximately 37–57 percent reduction in risk. Moreover, subjects with UIC between 100 and 199 *μ*g l^−1^ had a decreased risk of thyroid nodules in model 1 and 2 (OR = 0.75, 95% CI, 0.58–0.97; OR = 0.75, 95% CI, 0.58–0.97, respectively). However, there was no significant difference of risk in thyroid nodules between subjects with high UIC (≥400 *μ*g l^−1^) and low UIC (<100 *μ*g l^−1^). Furthermore, intake of iodized salt was inversely associated with risk of thyroid nodules, with approximately 69–77 percent reduction in risk.

**Conclusion:**

The relationship between UIC and the risk of thyroid nodules is U-shaped. Consumption of noniodized salt is an independent risk factor of thyroid nodules.

## 1. Introduction

The thyroid nodules are a discrete lesion within the thyroid gland that is radiologically distinct from the surrounding thyroid parenchyma [1]. Thyroid nodules are a common clinical thyroid disease [2]. Epidemiologic studies have shown that the prevalence of thyroid nodules was approximately 4%–7% as determined by palpation [3]. Meanwhile, the prevalence of thyroid nodules was 19%–67% on high-resolution ultrasound [4]. Approximately 7%–15% of thyroid nodules are malignant [3, 5]. Some environmental factors, such as age, iodine intake, gender, lifestyle, and radiation history of the head and neck, could affect the risk of thyroid nodules [6–8]. Iodine is an essential element required for the synthesis of thyroid hormones, thyroxine and triiodothyronine. Thyroid hormone disorders could be induced due to both insufficient and excessive iodine intakes. Some studies revealed that low iodine intake could increase the risk of thyroid nodules [9, 10]. On the other hand, a few studies have shown that excessive iodine intake may also lead to development of thyroid nodules. Whether excessive iodine intake is involved in the increase of thyroid nodules occurrence remains unclear. The aim of this study was to explore whether iodine nutrition is associated with the risk of thyroid nodules and to investigate the effects of salt iodine on the prevalence of thyroid nodules among adults.

## 2. Methods

### 2.1. Subjects

This study was conducted in Zhejiang Province, which is located on the east China coast. The recruit strategy of study participants was performed as follows. Firstly, one district and one county were selected randomly from Zhejiang Province. Secondly, two subdistricts or towns were selected randomly from each selected district or county. Thirdly, residents of these subdistricts or towns were recruited based on age and gender ratio of the population census in 2010 [11]. Finally, our study included 2,710 participants, of which 1,395 were from the district and 1,315 were from the county. The main inclusions were participants who aged ≥18 years and lived for ≥5 years at local residence. The exclusion criteria were participants who received contrast-enhanced ultrasonography with iodine-containing contrast agent, or those who have taken the drug of ethylamiodarone in the past three months, or those who were pregnant. Our study was conducted from December 2015 through July 2016. Ethical approval was obtained from the ethics committee of the Zhejiang Provincial Centre for Disease Prevention and Control, and all participants have provided written informed consent.

### 2.2. Lifestyle and Examination Data

The subjects completed questionnaires regarding lifestyle and iodine nutrition information through face-to-face interviews by well-trained investigators. Demographic characteristics, iodine nutrition status, personal and family history of thyroid disease, general medical history, and smoking status of interviewees were documented. Furthermore, information about types of salt used in households, including iodized salt or noniodized salt, was collected. The quality of questionnaire completion was checked daily. If blank entries were found, we would further gather the missing information through phone interviews. Additionally, anthropometric and physical examinations were also performed by professional public health doctors. BMI was calculated as weight (kg)/height (m)^2^. Thyroid status was examined to detect thyroid nodules by portable ultrasound machine (GE, LOGIQ*α*100, Boston, MA, USA) with a 7.5-MHZ, 60-mm transducer.

### 2.3. Iodine Concentration and Biochemistry Data

Each participant provided 5 mL spot urine and 10 mL venous blood sample, which were immediately placed at −20°C before being sent to the laboratory for further analysis. Urinary iodine concentration (UIC) was measured using the spectrophotometer method (WS/T 107-2006) [12]. We applied the reference material (GBW09108, GBW9109, and GBW9110) from the Centre for Disease Control (CDC) for China for quality control. The values of the reference materials were 70.8 ± 9.0 *μ*g l^−1^, 143 ± 10 *μ*g l^−1^, and 224 ± 14 *μ*g l^−1^, with an interassay CV of 2.3%, 2.5%, and 2.4%, and an intra-assay CV of 2.7%, 1.4%, and 2.3%, respectively. According to the recommended iodine nutrition status evaluation criteria of the WHO [13], iodine deficiency was indicated by median urinary iodine (MUI) < 100 *µ*g l^−1^, adequate iodine nutrition by 100 *µ*g l^−1^ ≤ MUI ≤ 199 *µ*g l^−1^, above requirements by 200 *µ*g l^−1^ ≤ MUI ≤ 299 *µ*g l^−1^, and excessive by MUI ≥ 300 *µ*g l^−1^.

Serum levels of thyroid-stimulating hormone (TSH), thyroid peroxidase antibody (TPOAb), and thyroglobulin antibody were measured for each subject by using the immunochemiluminescent method from a Cobas 601 analyzer (Roche Diagnostic, Switzerland). Free thyroxine (FT4) and free triiodothyronine (FT3) levels were measured only if TSH was outside the reference range (0.27–4.2 mIU l^−1^).The repeatability of serum assays of TSH, FT4, FT3, TPOAb, and TGAb was ensured by intra-assay coefficients of variation (CV) of 1.1%–6.3% and interassay CV of 1.9%–9.5% [14]. The thyroid functional status for each participant was identified based on the diagnostic criteria for thyroid disorders [14].

Fasting blood glucose (FBG), blood glucose after 2 hour, 75-gram oral glucose tolerance test (OGTT-2h), triglyceride (TG), total cholesterol (TC), low-density lipoprotein cholesterol (LDL-C), high-density lipoprotein cholesterol (HDL-C), and uric acid (UA) were determined for all participants by using an automatic chemistry analyzer (Mindray, BS-180, Shenzhen, China), and hemoglobin A1c (HbA1c) was measured by an HbA1c analyzer (Bio-Rad, VARIANT II Hemoglobin Analyzer, CA, USA). The measuring methods were reported in detail previously [15].

### 2.4. Statistical Analysis

We used the statistical analysis system (SAS) software (version 9.4, Cary, NC, USA) for statistical analysis. Continuous variables which were normally distributed according to the Shapiro–Wilk test and categorical variables were described as mean (SD) and percentages, respectively. The *t*-test and chi-square test were applied to test the difference between subjects with and without thyroid nodules in terms of categorical and continuous variables. Other continuous variables that were not normally distributed were presented as median (interquartile range (IQR)), and we used Wilcoxon test to assess the differences in those.

We further examined the nonlinear or linear associations between UIC and the prevalence of thyroid nodules as follows. Firstly, UIC was cut into ten groups according to decile. Secondly, we calculated the MUI of each group as exposure and the prevalence of thyroid nodules of each group as response. Finally, the quadratic, linear, and spline fitted GLM models were fitted to examine the associations. To exhibit the relationship more clearly, we generated expose-response curves to visualize the effects of UIC on the prevalence of thyroid nodules.

Logistic regression models were used to estimate the effect of UIC, and types of salt on thyroid nodules were stratified by gender. In order to reduce confounders, we only included these variables which have significant effect on the risk of thyroid nodules according to the result of univariate analysis. The base statistical mode (model 1) was adjusted for demographic characteristics including gender (male vs. female), age (year, continuously), education (illiteracy/primary school, junior middle school, high school/technical secondary school, and university above), and profession (physical labor, agricultural labor, mental labor, and others). The associations were assessed by three additional models (model 2, 3, and 4). Model 2 additionally corrected for hypertension (yes vs. no) based on model 1. In model 3, some physical examination and biochemistry variables, such as BMI (mg dl^−1^,continuously), waist circumference (cm, continuously), FBG (mmol l^−1^, continuously), OTGG-2h (mmol l^−1^, continuously), HbA1c (year, continuously), TC (mmol l^−1^,continuously), LDL-C (mmol l^−1^,continuously), and UA (mmol l^−1^,continuously), were included as covariates in addition to confounding factors in model 2. Model 4 further adjusted thyroid hormone including TSH (mIU l^−1^, continuously) and TGAb (IU l^−1^, continuously). The linear combination was used to calculate the estimates for different genders [16]. Results are expressed as odds ratio (OR) and 95% confidence interval (95% CI).

## 3. Results

The prevalence of thyroid nodules and MUI was 15.5% (420/2710) and 158.0 (134.2) *μ*g l^−1^ among all adults, respectively. The MUIs of subjects with and without thyroid nodules were 122.9 (110.3) *μ*g l^−1^and 164.0(134.6) *μ*g l^−1^, respectively, and subjects with thyroid nodules had lower MUI than subjects without thyroid nodules (*p* < 0.01). The correlation coefficient between the reported salt intake with the corresponding UIC is 0.4 with a significant *p* value (*p* < 0.01).

Subjects who belong to the category of female, older, low educational level, agricultural worker, hypertensive patients, consumed noniodized salt, and low UIC had a higher prevalence of thyroid nodules (*p* < 0.01). Compared with subjects without thyroid nodules, those with thyroid nodules reported a significantly higher level of BMI and waist circumference and higher values of FBG, OTGG-2h, HbA1c, TC, LDL, and TGAb, as well as lower values of UA and TSH. Meanwhile, we found no significant differences between participants with and without thyroid nodules in terms of income, region, smoke, passive smoke, family history of thyroid diseases, heart rate, TG, HDL, thyroid functional status, and TPOAb (Table 1). From Table 1, we could see that the prevalence of thyroid nodules gradually increased with age (*p* < 0.01).

**Figure 1:**
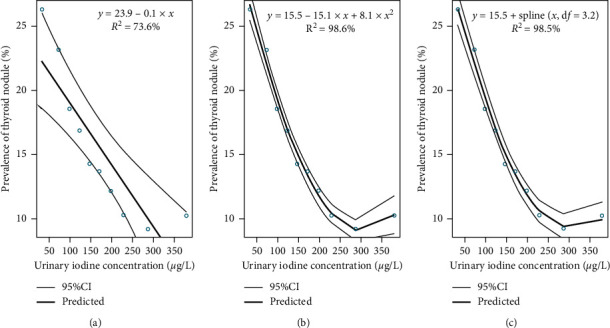
The nonlinear and linear associations between urinary iodine concentration and prevalence of thyroid nodule: (a) the linear relationship between urinary iodine concentration and prevalence of thyroid nodule was observed with adjusted *R*^2^ = 73.6%; (b) the quadratic curve existed between urinary iodine concentration and prevalence of thyroid nodule with adjusted *R*^2^ = 98.6%; (c) the nonlinear relationship between urinary iodine concentration and prevalence of thyroid nodule was observed by using spline function with adjusted *R*^2^ = 98.5%.

Significant quadratic, spline, and linear associations were discovered between UIC and the prevalence of thyroid nodules, respectively (*p* < 0.01). Moreover, larger adjusted *R* squares were observed in quadratic and spline fitted GLM models than that in the linear model. From Figure 1, we could find the highest prevalence of thyroid nodules in the low-UIC group. Given the results, the iodine deficiency group (low UIC) was defined as reference in the logistic regression model. Meanwhile, to investigate the effect of excessive UIC on thyroid nodules, excessive UIC was subdivided into 300–399, 400–499, and ≥ 500 *μ*g l^−1^ groups. Next, we would fit two series multiple logistic models with effects of UIC on thyroid nodules, one of which classified UIC according to the iodine nutritional status evaluation criteria of the WHO (Table 2), and the other of which adopted the UIC as 6 groups with subdivided excessive UIC (≥300 *μ*g l^−1^) into 300–399, 400–499, and ≥ 500 *μ*g l^−1^ groups (Table 3).

Tables 2 and 3 present the results of the four multiple logistic models regarding the association between UIC and thyroid nodules as ORs, 95% CIs, and *p* values. For all adults, subjects with UIC varying from 200 *μ*g l^−1^ to 399 *μ*g l^−1^ had a lower risk of thyroid nodules compared with those with low UIC (<100 *μ*g l^−1^) in all four models, with approximately 37–57 percent reduction in risk. Moreover, subjects with UIC between 100 *μ*g l^−1^ and 199 *μ*g l^−1^ had a decreased risk of thyroid nodules in model 1 and 2 (OR = 0.75, 95% CI, 0.58–0.97; OR = 0.75, 95% CI, 0.58–0.97, respectively). However, there was no significant difference regarding risk in thyroid nodules between subjects with high UIC (≥400 *μ*g l^−1^) and low UIC (<100 *μ*g l^−1^) in all four models. Separate analysis was performed stratified by gender. Similar associations were observed in the female group. Males with UIC between 200 *μ*g l^−1^ and 299 *μ*g l^−1^ had an inverse association with risk of thyroid nodules in model 1 and 2 (OR = 0.55, 95% CI, 0.34–0.91; OR = 0.55, 95% CI, 0.34–0.91, respectively).

Furthermore, intake of iodized salt was inversely associated with risk of thyroid nodules in all four models, with approximately 69–77 percent reduction in risk. Similar associations were observed in male and female groups (Table 4).

## 4. Discussion

In this study, significant association was observed between iodine nutrition and thyroid nodules among 2,710 participants in Zhejiang Province, China. We found that adults who had a low or high UIC or with consumption of noniodized salt had a higher risk of thyroid nodules, suggesting that adequate iodine nutrition may decrease the risk of thyroid nodules.

The prevalence of thyroid nodules in adults of Zhejiang Province was 15.5%. It is slightly lower than that of mainland China (22.8%) [17], France (34.7%) [18], and Germany (23.4%) [19]. Moreover, it is partly in line with that in Korea (13.4%) [20] and Brazil (17.0%) [21]. Many epidemiological studies [22–24] have presented that the prevalence of thyroid nodules increases with age. The mechanism may be that the thyroid will undergo degenerative changes with age, resulting in development of nodules [24].

In our study, we found that the relationship of UIC and the risk of thyroid nodules presented a U-shaped curve with an increase in risk from both low- and high-iodine intake levels in both univariate and multivariate models. It was consistent with other studies [25–27]. Iodine deficiency could contribute to elevating the risk of thyroid nodules; several mechanisms may be involved in this association. Firstly, iodine deficiency leads to the increase of TSH due to the feedback regulation of the hypothalamus-pituitary-thyroid axis. After a period of time, thyroid follicular cell compensatory hyperplasia and hypertrophy form nodules [23]. Secondly, previous studies found that iodine deficiency could cause the formatting thyroid nodules by generating autonomous thyrocyte cluster via the promotion of thyrocyte cell growth and DNA mutagenesis [28]. Moreover, it is well known that iodized salt is the main contribution to dietary iodine intake [29]. According to previous studies [29], the dietary iodine intake from iodized salt was 250.03 *μ*g d^−1^, which accounted for 73.45% of total dietary iodine indicating that subjects would lose 73.45% of dietary iodine if they consumed noniodized salt. Therefore, subjects who consumed noniodized salt could lead to a low UIC. Likewise, we also found that noniodized salt increased the risk of thyroid nodules. These results confirmed the link of noniodized salt intake-low UIC-high risk of thyroid nodules, which is consistent with previous studies [9, 23]. Furthermore, we found that 25.8% of adults had iodine deficiency although the MUI results indicated adequate iodine states. Therefore, full iodized salt exposure still remains essential for all subjects. Meanwhile, iodine deficiency may result in higher impact on female than male in part due to the reason that females were more sensitive to levels of hormones and different levels of iodine intake [30].

However, so far, there is no exact mechanism regarding excessive iodine leading to thyroid nodules [23, 30]. Meanwhile, few studies have been conducted to explore the association between excessive iodine intake and thyroid nodules. Previous studies [9, 25, 31, 32] have reported that no significant association were found between excessive iodine intake and thyroid nodules with controlled confound factors. In this study, we subdivided excessive iodine intake into 300–399, 400–499, and ≥ 500 *μ*g l^−1^ groups to explore this association, and we found that the risk of thyroid nodules was not significantly different when comparing low UIC with higher UIC which was greater than 400 *μ*g l^−1^ after adjusted confounder. It means the risk of thyroid nodules may increase when UIC was > 400 *μ*g l^−1^. This finding is similar to the study conducted in Shanghai [30] which borders Zhejiang Province. However, the relatively small sample size of subjects who had UIC > 400 *μ*g l^−1^ may be a limitation to fully demonstrate this association. Thus, further studies were needed to provide direct evidence of whether excessive iodine intake increases the risk of thyroid nodules and precisely define the threshold of excessive iodine intake.

Some limitations of this study are as follows. Firstly, we enrolled insufficient subjects who were exposed to excessive iodine; therefore, there was not enough power to fully demonstrate the association between thyroid nodules and high UIC. Secondly, our study belongs to cross-section studies, which could not clarify whether the occurrence of thyroid nodules cause reduction in UIC or the low UIC leads to increase the risk of thyroid nodules. Thus, observational studies with focuses on subjects with high iodine exposure are warranted in future.

## 5. Conclusions

In summary, our study revealed the association between iodine nutrition and the risk of thyroid nodules. The relationship between UIC and the risk of thyroid nodules is U-shaped. Consumption of noniodized salt is an independent risk factor of thyroid nodules.

## Figures and Tables

**Table 1:** Summary statistics of variables between nonthyroid nodule and thyroid nodule groups.

Variables	Nonthyroid nodule (*n* = 2290)	Thyroid nodule (*n* = 420)	Total (*n* = 2710)	*p*
Gender^a^				0.00
Female	1108 (81.7)	249 (18.4)	1357 (50.1)	
Male	1182 (87.4)	171 (12.6)	1353 (49.9)	
Age (year^b^)	42.40 (15.8)	50.09 (15.5)	43.0 (25.0)	0.00
Age group^a^				0.00
18–29	608 (91.8)	54 (8.2)	662 (24.4)	
30–39	478 (88.4)	63 (11.7)	541 (20.0)	
40–49	487 (85.0)	86 (15.0)	573 (21.1)	
50–59	340 (79.1)	90 (20.9)	430 (15.9)	
60–69	223 (75.1)	74 (24.9)	297 (11.0)	
≥70	154 (74.4)	53 (25.6)	207 (7.6)	
Education^a^				
Illiteracy/primary school	521 (79.4)	135 (20.6)	656 (24.2)	
Junior middle school	687 (83.5)	136 (16.5)	823 (30.4)	
High school/technical secondary school	476 (87.8)	66 (12.2)	542 (20.0)	
University/college/graduate student	606 (88.0)	83 (12.0)	689 (25.4)	
Profession^a^				0.00
Physical labor	932 (84.4)	172 (15.6)	1104 (40.7)	
Agricultural labor	275 (76.0)	87 (24.0)	362 (13.4)	
Mental labor	694 (88.5)	90 (11.5)	784 (28.9)	
Others	389 (84.6)	71 (15.4)	460 (17.0)	
Income, thousand yuan^a^				0.06
<10	252 (88.1)	34 (11.9)	286 (10.6)	
10–29	367 (82.8)	76 (17.2)	443 (16.4)	
30–49	626 (82.0)	137 (18.0)	763 (28.2)	
50–99	636 (85.6)	107 (14.4)	743 (27.4)	
≥100	409 (86.1)	66 (13.9)	475 (17.5)	
Region^a^				0.41
County	1119 (85.1)	196 (14.9)	1315 (48.5)	
District	1171 (83.9)	224 (16.1)	1395 (51.5)	
Smoke^a^				0.94
No	1874 (84.5)	343 (15.5)	2217 (81.8)	
Yes	416 (84.4)	77 (15.6)	493 (18.2)	
Passive smoke^a^				0.82
No	1648 (84.6)	300 (15.4)	1948 (71.9)	
Yes	642 (84.3)	120 (15.7)	762 (28.1)	
Family history of thyroid diseases^a^				0.60
No	2194 (84.6)	400 (15.4)	2594 (95.7)	
Yes	96 (82.8)	20 (17.2)	116 (4.3)	
Hypertension^a^				0.00
No	1724 (86.1)	278 (13.9)	2002 (73.9)	
Yes	566 (79.9)	142 (20.1)	708 (26.1)	
BMI (kg/m^2b^)	23.4 (3.4)	24.2 (3.2)	23.6 ± 3.4	0.00
Waist circumference (cm^b^)	80.8 (9.5)	84.7 (8.8)	81.4 ± 9.5	0.00
Heart rate (times/minute^b^)	79.7 (11.7)	80.2 (11.2)	79.8 ± 11.6	0.41
FBG (mmol l^−1b^)	5.5 (1.5)	5.7 (1.3)	5.5 ± 1.5	0.00
OGTT-2h (mmol l^−1b^)	5.9 (2.2)	6.3 (2.4)	5.9 ± 2.2	0.00
HbA1c^b^	5.4 (1.1)	5.6 (0.8)	5.5 ± 1.0	0.00
TG (mmol l^−1b^)	1.6 (2.9)	1.6 (1.4)	1.6 ± 2.7	0.96
TC (mmol l^−1b^)	4.6 (1.3)	5.0 (1.1)	4.7 ± 1.3	0.00
LDL-C (mmol l^−1b^)	2.8 (0.8)	3.1 (0.9)	2.9 ± 0.8	0.00
HDL-C (mmol l^−1b^	1.6 (0.5)	1.6 (0.4)	1.6 ± 0.5	0.32
UA (mmol l^−1b^)	297.1 (169.8)	285.1 (84.7)	295.2 ± 159.7	0.03
TSH (mIU l^−1c^)	2.2 (1.8)	2.0 (1.4)	2.2 (1.73)	0.00
Overt hyperthyroidism^a^				1.00
No	2282 (84.5)	419 (15.5)	2701 (99.7)	
Yes	8 (88.9)	1 (11.1)	9 (0.3)	
Subclinical hyperthyroidism^a^				0.05
No	2287 (84.6)	417 (15.4)	2704 (99.8)	
Yes	3 (50)	3 (50)	6 (0.2)	
Overt hypothyroidism^a^				1.00
No	2275 (84.5)	418 (15.5)	2693 (99.4)	
Yes	15 (88.2)	2 (11.8)	17 (0.6)	
Subclinical hyperthyroidism^a^				0.05
No	1989 (84.5)	366 (15.5)	2355 (86.9)	
Yes	314 (88.5)	41 (11.5)	355 (13.1)	
TPOAb (IU l^−1c^)	11.5 (5.7)	11.8 (5.7)	11.6 (5.7)	0.94
TGAb (IU l^−1c^)	12.5 (6.7)	13.2 (7.3)	12.6 (7.0)	0.00
Iodized salt^a^				0.00
No	706 (71.8)	277 (28.2)	983 (36.3)	
Yes	1584 (91.7)	143 (8.3)	1727 (63.7)	
UIC (*μ*g l^−1C^)	164.0 (134.6)	122.9 (110.3)	158 (134.2)	0.00
UIC (*μ*g l^−1a^)				0.00
<100	541 (23.6)	158 (37.6)	699 (25.8)	
100–199	917 (40)	165 (39.3)	1082 (39.9)	
200–299	530 (23.1)	63 (15)	593 (21.9)	
≥300	302 (13.2)	34 (8.1)	336 (12.4)	

^a^Expression with the number (proportion %), tested with the chi-square test. ^b^Expression with mean (SD), tested with the *t*-test. ^c^Expression with median (IQR), tested with the Wilcoxon method.

**Table 2:** Effect of urinary iodine concentration on thyroid nodule according to the iodine nutritional status evaluation criteria of the WHO.

Group	UIC (*μ*g l^−1^)	Model 1^a^		Model 2^b^		Model 3^c^		Model 4^d^	
		OR (95% CI)	*p*	OR (95% CI)	*p*	OR (95% CI)	*p*	OR (95% CI)	*p*
All	<100	1.00		1.00		1.00		1.00	
	100–199	0.75 (0.58–0.97)	0.03	0.75 (0.58–0.97)	0.03	0.78 (0.61–1.01)	0.06	0.79 (0.61–1.03)	0.08
	200–299	0.54 (0.39–0.75)	0.00	0.54 (0.39–0.75)	0.00	0.62 (0.44–0.87)	0.01	0.63 (0.45–0.88)	0.01
	≥300	0.50 (0.34–0.76)	0.00	0.51 (0.34–0.77)	0.00	0.61 (0.40–0.92)	0.02	0.62 (0.41–0.94)	0.03

Male	<100	1.00		1.00		1.00		1.00	
	100–199	0.83 (0.56–1.23)	0.34	0.82 (0.55–1.22)	0.33	0.84 (0.56–1.25)	0.38	0.84 (0.56–1.25)	0.39
	200–299	0.55 (0.34–0.91)	0.02	0.55 (0.34–0.91)	0.02	0.63 (0.38–1.04)	0.07	0.63 (0.38–1.04)	0.07
	>300	0.67 (0.37–1.20)	0.18	0.68 (0.38–1.21)	0.19	0.84 (0.47–1.52)	0.57	0.85 (0.47–1.53)	0.58

Female	<100	1.00		1.00		1.00		1.00	
	100–199	0.71 (0.51–0.98)	0.04	0.71 (0.51–0.98)	0.04	0.75 (0.54–1.05)	0.09	0.77 (0.55–1.07)	0.12
	200–299	0.54 (0.35–0.83)	0.01	0.54 (0.35–0.84)	0.01	0.62 (0.40–0.97)	0.04	0.63 (0.40–0.98)	0.04
	≥300	0.39 (0.22–0.70)	0.00	0.40 (0.22–0.70)	0.00	0.46 (0.25–0.82)	0.01	0.47 (0.26–0.85)	0.01

^a^Adjusted for gender, age (continuous variable), education, and profession.^b^Additionally adjusted for hypertension.^c^Additionally adjusted for BMI, waist circumference, FBG, OTGG-2h, HbA1c, TC, LDL-C, and UA.^d^Additionally adjusted for TSH and TGAb.

**Table 3:** Effect of urinary iodine concentration on thyroid nodule according to the iodine nutritional status evaluation criteria of the WHO.

Group	UIC (*μ*g l^−1^)	Model 1^a^		Model 2^b^		Model 3^c^		Model 4^d^	
		OR (95% CI)	*p*	OR (95% CI)	*p*	OR (95% CI)	*p*	OR (95% CI)	*p*
All	<100	1.00		1.00		1.00		1.00	
	100–199	0.75 (0.58–0.97)	0.03	0.75 (0.58–0.97)	0.03	0.78 (0.61–1.01)	0.06	0.79 (0.61–1.03)	0.08
	200–299	0.54 (0.39–0.75)	0.00	0.54 (0.39–0.75)	0.00	0.62 (0.44–0.87)	0.01	0.62 (0.45–0.87)	0.01
	300–399	0.43 (0.24–0.76)	0.00	0.43 (0.24–0.76)	0.00	0.52 (0.29–0.92)	0.03	0.53 (0.30–0.94)	0.03
	400–499	0.42 (0.18–1.00)	0.05	0.42 (0.18–1.00)	0.05	0.52 (0.21–1.25)	0.14	0.52 (0.22–1.26)	0.15
	≥500	0.73 (0.39–1.36)	0.32	0.73 (0.39–1.37)	0.33	0.85 (0.45–1.60)	0.61	0.88 (0.47–1.67)	0.71

Male	<100								
	100–199	0.83 (0.56–1.23)	0.34	0.82 (0.55–1.22)	0.33	0.84 (0.56–1.25)	0.39	0.84 (0.56–1.25)	0.39
	200–299	0.55 (0.34–0.91)	0.02	0.55 (0.34–0.91)	0.02	0.63 (0.38–1.04)	0.07	0.63 (0.38–1.05)	0.07
	300–399	0.57 (0.26–1.26)	0.16	0.57 (0.26–1.26)	0.17	0.74 (0.33–1.65)	0.46	0.74 (0.33–1.66)	0.47
	400–499	0.31 (0.07–1.34)	0.12	0.31 (0.07–1.36)	0.12	0.39 (0.09–1.74)	0.22	0.39 (0.09–1.73)	0.22
	≥500	1.29 (0.55–3.01)	0.56	1.31 (0.56–3.05)	0.53	1.48 (0.63–3.48)	0.37	1.50 (0.64–3.54)	0.35

Female	<100								
	100–199	0.71 (0.51–0.98)	0.04	0.71 (0.51–0.98)	0.04	0.75 (0.54–1.05)	0.09	0.77 (0.55–1.07)	0.12
	200–299	0.54 (0.35–0.83)	0.01	0.54 (0.35–0.84)	0.01	0.62 (0.40–0.97)	0.04	0.63 (0.40–0.98)	0.04
	300–399	0.33 (0.15–0.76)	0.01	0.34 (0.15–0.76)	0.01	0.38 (0.16–0.86)	0.02	0.39 (0.17–0.89)	0.03
	400–499	0.52 (0.18–1.54)	0.24	0.52 (0.17–1.54)	0.24	0.63 (0.21–1.88)	0.40	0.63 (0.21–1.90)	0.41
	≥500	0.42 (0.16–1.10)	0.08	0.42 (0.16–1.11)	0.08	0.50 (0.19–1.32)	0.16	0.53 (0.20–1.39)	0.20

^a^Adjusted for gender, age (continuous variable), education, and profession.^b^Additionally adjusted for hypertension.^c^Additionally adjusted for BMI, waist circumference, FBG, OTGG-2h, HbA1c, TC, LDL-C, and UA.^d^Additionally adjusted for TSH and TGAb.

**Table 4:** Effect of iodized salt intake on thyroid nodule.

Group	Iodized salt	Model 1^a^		Model 2^b^		Model 3^c^		Model 4^d^	
		OR (95% CI)	*p*	OR (95% CI)	*p*	OR (95% CI)	*p*	OR (95% CI)	*p*
All	No	1.00		1.00		1.00		1.00	
	Yes	0.25(0.19–0.31)	0.00	0.25(0.19–0.31)	0.00	0.29(0.22–0.37)	0.00	0.29(0.23–0.37)	0.00

Male	No	1.00		1.00		1.00		1.00	
	Yes	0.23(0.16–0.32)	0.00	0.23(0.16–0.33)	0.00	0.27(0.19–0.38)	0.00	0.27(0.19–0.39)	0.00

Female	No	1.00		1.00		1.00		1.00	
	Yes	0.26(0.19–0.36)	0.00	0.26(0.19–0.35)	0.00	0.30(0.22–0.42)	0.00	0.31(0.22–0.43)	0.00

^a^Adjusted for gender, age (continuous variable), education, and profession.^b^Additionally adjusted for hypertension.^c^Additionally adjusted for BMI, waist circumference, FBG, OTGG-2h, HbA1c, TC, LDL-C, and UA.^d^Additionally adjusted for TSH and TGAb.

## Data Availability

The data belong to the funders and are not available to the public in order to protect the patient privacy.
